# Identification of a Novel Polymorphism in X-Linked Sterol-4-Alpha-Carboxylate 3-Dehydrogenase (*Nsdhl*) Associated with Reduced High-Density Lipoprotein Cholesterol Levels in I/LnJ Mice

**DOI:** 10.1534/g3.113.007567

**Published:** 2013-10-01

**Authors:** David J. Bautz, Karl W. Broman, David W. Threadgill

**Affiliations:** * Department of Genetics, North Carolina State University, Raleigh, North Carolina 27695; † Department of Biostatistics and Medical Informatics, University of Wisconsin–Madison, Madison, Wisconsin 53706

**Keywords:** QTL, genomics, HDL cholesterol, Nsdhl

## Abstract

Loci controlling plasma lipid concentrations were identified by performing a quantitative trait locus analysis on genotypes from 233 mice from a F2 cross between KK/HlJ and I/LnJ, two strains known to differ in their high-density lipoprotein (HDL) cholesterol levels. When fed a standard diet, HDL cholesterol concentration was affected by two significant loci, the *Apoa2* locus on Chromosome (Chr) 1 and a novel locus on Chr X, along with one suggestive locus on Chr 6. Non-HDL concentration also was affected by loci on Chr 1 and X along with a suggestive locus on Chr 3. Additional loci that may be sex-specific were identified for HDL cholesterol on Chr 2, 3, and 4 and for non-HDL cholesterol on Chr 5, 7, and 14. Further investigation into the potential causative gene on Chr X for reduced HDL cholesterol levels revealed a novel, I/LnJ-specific nonsynonymous polymorphism in *Nsdhl*, which codes for sterol-4-alpha-carboxylate 3-dehydrogenase in the cholesterol synthesis pathway. Although many lipid quantitative trait locus have been reported previously, these data suggest there are additional genes left to be identified that control lipid levels and that can provide new pharmaceutical targets.

Coronary artery disease is the leading cause of death in the United States (Hoyert and Xu 2012). Multiple studies have consistently shown an inverse correlation between the level of high-density lipoprotein (HDL) cholesterol and the incidence of coronary artery disease (Di Angelantonio *et al.* 2009; Landmesser 2012). Identification of genes that control the level of HDL cholesterol are actively being pursued as potential therapeutic targets; however, only a limited number of proteins have been targeted thus far, and there remain few drugs that are capable of increasing HDL.

Cholesterol level is a complex trait controlled by multiple loci, which is supported by the results of a number of human and mouse genetic studies (Wang *et al.* 2005) with most studies in humans show a heritability of between 40 and 60% (Peacock *et al.* 2001). To date, more than 25 mouse crosses using 24 strains have been performed to examine the genetic basis of cholesterol levels, resulting in the detection of more than 40 quantitative trait loci (QTL) (Wang and Paigen 2005; Stylianou *et al.* 2006; Wergedal *et al.* 2007; Stylianou *et al.* 2008; Su *et al.* 2009a; Su *et al.* 2010; Leduc *et al.* 2012). Most QTL identified in mice show conserved synteny with loci controlling cholesterol levels detected by genome-wide association studies in humans (Leduc *et al.* 2011), validating the use of mice for studying the genetic control of cholesterol levels. Although genome-wide association studies have found a number of common variants in genes known to play a role in cholesterol level, the effect sizes of the reported associations is only 5–10% cumulatively, suggesting there are additional loci that remain to be identified (Su *et al.* 2009b).

Here, using an intercross between KK/HlJ (KK) and I/LnJ (I/L) mouse strains reared on standard mouse chow, a cross not previously evaluated for cholesterol levels, we report the confirmation of previously reported loci and the identification of additional, novel QTL controlling HDL and non-HDL cholesterol levels. Several loci were found that may be sex-specific, and a candidate gene for a novel HDL level QTL on Chromosome (Chr) X was investigated. We sequenced *Nsdhl*, which codes for sterol-4-alpha-carboxylate 3-dehydrogenase in the cholesterol biosynthesis pathway and which is found within the HDL level QTL on Chr X, from KK and I/L strains. A nonsynonymous polymorphism in a highly conserved residue of *Nsdhl* was identified in I/L mice that has not been previously reported in any other mouse strain, suggesting that *Nsdhl* is the causal gene for the HDL level QTL on Chr X.

## Materials and Methods

### Mice

I/LnJ and KK/HlJ inbred strains were obtained from The Jackson Laboratory (Bar Harbor, ME). Reciprocal crosses between I/L and KK mice produced a KKIF1 and IKKF1 mice that were intercrossed to produce a KKIF2 population of 233 mice (127 males, 106 females; Supporting Information, Table S1). All mice were weaned at 21 d of age onto standard mouse chow (LabDiets 5015; St. Louis, MO). Mice were housed in a climate-controlled facility with a 12-hr light-dark cycle and allowed *ad libitum* access to food and water. All animal protocols were reviewed and approved by the Institutional Animal Care and Use Committee at North Carolina State University.

### Plasma lipid analysis

Blood from the tail vein was collected from 12- to 14-wk-old mice in tubes containing ethylenediamine tetraacetic acid and spun at 10,000*g* for 5 min. Plasma was frozen at −20° until tested. Mice were not fasted prior to blood collection. Plasma HDL and total cholesterol were measured using a VT350 Automatic Chemical Analyzer (Johnson & Johnson; Indianapolis, IN). Non-HDL cholesterol was calculated as the difference between the total cholesterol and HDL cholesterol.

### Genotyping

DNA was extracted from liver using a Maxwell 16 System (Promega; Madison, WI). A custom single-nucleotide polymorphism (SNP) array was designed with 137 SNPs evenly spaced across the genome. SNPs were genotyped using the VeraCode GoldenGate Genotyping Assay (Illumina, San Diego, CA) and read on the BeadXpress Reader. Data normalization and genotype calls were performed using GenomeStudio software.

### QTL analysis

QTL mapping was performed using R/QTL (version 1.25−15; http://www.rqtl.org) (Broman *et al.* 2003). HDL and non-HDL cholesterol values were log base 2 transformed to obtain a normal distribution. Coordinates for markers were based on the genetic map previously described (Cox *et al.* 2009). Main effect QTLs were computed over 1-cM increments across the entire genome using composite interval mapping. The direction of the crosses was not recorded, and as a result, there were 24 females that we were unable to definitively determine the direction of the cross from which they originated. Cross direction is important for the treatment of Chr X in interval mapping. We performed QTL analysis in two ways: we assigned these 24 females to one of the two cross directions at random, and we modified the likelihood function to allow for the uncertainty in cross direction. No difference was seen between the two analyses. A large QTL was detected on Chr 1 likely due to the presence of well-described polymorphisms at the *Apoa2* locus (Wang *et al.* 2004). To account for this known QTL, the genotypes of the nearest SNP to *Apoa2* (rs13476229) were added as an additive covariate and the QTL analysis was performed again. To aid in identifying QTL that may be sex-specific, we performed additional analyses in each sex separately. Interval estimates of QTL location were derived as approximate 95% Bayesian credible intervals. All significant and suggestive QTL were combined in a multi-locus model with the percentage of the variation explained by each QTL computed with regression analysis. Significant (*P* < 0.05) and suggestive (*P* < 0.63) logarithm of the odds ratio (LOD) threshold levels were chosen because they are the widely accepted cutoffs (Lander and Kruglyak 1995) and were calculated using 1,000 permutations for the autosomes (Churchill and Doerge 1994) and 16,803 permutations for Chr X, as the number of degrees of freedom is different for Chr X and thus many more permutation replicates are required to achieve a threshold of equivalent precision (Broman *et al.* 2006).

### *Nsdhl* sequencing

Total RNA was extracted from spleen (n = 2 each for I/L and KK) stored in RNAlater (Ambion) using Trizol (Invitrogen) according to the manufacturer’s protocol. Reverse-transcription polymerase chain reaction (PCR) was performed using a Supercript III RT-PCR kit and *Nsdhl*-specific primers (F: 5′-AAGGGTGGCGGGTGTTCAGC and R: 5′-TGGGGAGGGCACTGGGGAAC). The PCR product was ligated into cloning vector pCR2.1-TOPO using a TOPO TA cloning kit (Invitrogen), transfected into DH5α *Escherichia coli* (Invitrogen) and plated onto LB/Ampicillin plates overlayed with X-gal. Plasmids were purified from blue colonies and sequenced with sequencing primers M13R (5′-CAGGAAACAGCTATGAC) and M13F (5′-TGTAAAACGACGGCCAGT). Sequences were deposited into Genbank and assigned Genbank accession numbers KF356400 and KF356401.

### Statistical analysis

Total, HDL, and non-HDL cholesterol values are represented as mean ± SEM. One-way analysis of variance was performed to determine the statistical significance of the differences among the mean phenotype values of mice based upon genotype at specific markers. Data were analyzed with StatPlus Mac LE (AnalystSoft; Vancouver, BC, Canada).

## Results

### Localization of HDL and non-HDL QTL

**Figure 1:**
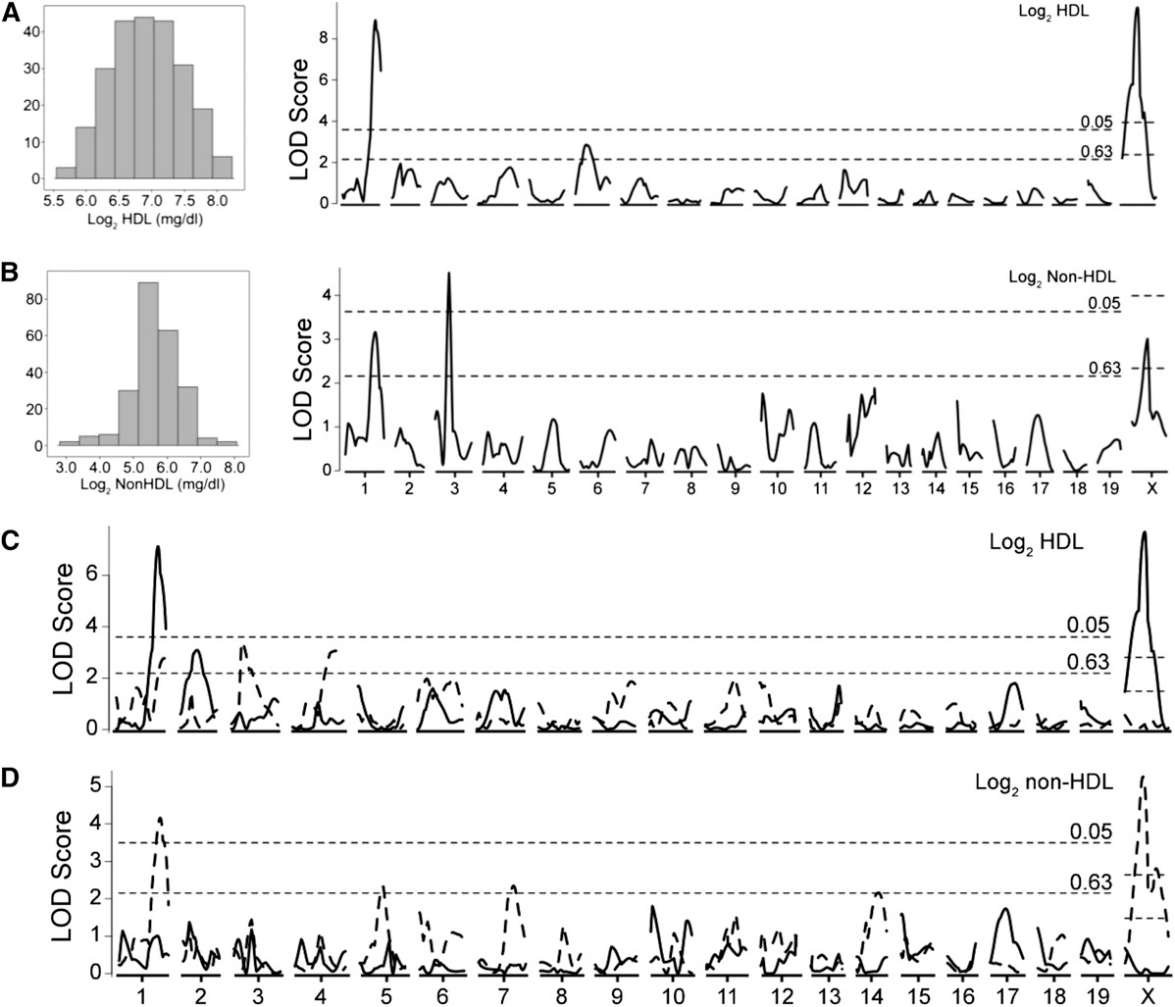
Genome-wide scan for HDL and non-HDL plasma level in 233 F2 progeny after 12−14 weeks on standard chow. Plasma HDL (A, C) and non-HDL (B, D) cholesterol values were log2-transformed before analysis. QTL analysis also was performed in males (n = 127, solid line) and females (n = 106, dashed line) separately (C, D). Chromosomes 1 through X are represented numerically along the x-axis with LOD scores plotted along the y-axis. Significant (*P* < 0.05) and suggestive (*P* < 0.63) levels were determined by 1000 permutations for the autosomes and 16,803 permutations for chromosome X.

Analysis of means and standard deviations for HDL and non-HDL cholesterol levels showed that there were no statistically significant differences between sexes for HDL cholesterol values (Table 1). However, there was a statistically significant difference in non-HDL values between males (n = 127) and females (n = 106). HDL and non-HDL values were log2 transformed resulting in a normalized distribution for each set of values (Figure 1, A and B). The log2-transformed data were used to localize QTL controlling HDL and non-HDL cholesterol levels (Figure 1, A and B; Table 2). For HDL levels, two significant and one suggestive QTL localized on Chr 1@74.7cM (*Cq1*), Chr 6@24.8cM and Chr X@38.4cM (*Hdlq84*). For all three HDL QTL, mice homozygous for the KK allele showed greater HDL levels, in an additive manner for *Cq1* and the Chr 6 QTL and a dominant manner for *Hdlq84*, compared with mice homozygous for the I/L allele. For non-HDL levels, one significant and two suggestive QTL were localized on Chr 1@67.6cM (*Nhdlq13*), Chr 3@32.4cM (*Nhdlq14*) and Chr X@38.3cM. For *Nhdlq14*, mice homozygous for the KK and I/L alleles did not differ in non-HDL but underdominance was suggested at this QTL because heterozygous mice had lower non-HDL levels than did KK and I/L homozygous mice. The HDL QTL on Chr 1@74.7cM has previously been identified in a number of other mouse crosses, including one involving KK mice (Suto *et al.* 1999) and is due to a specific polymorphism in the *Apoa2* gene (Wang *et al.* 2004). This result was expected as KK mice harbor the *Apoa2^b^* allele whereas I/L mice carry *Apoa2^a^*. Because of the very strong effect of the *Apoa2* locus, QTL analysis was repeated while we controlled for the effect of the *Apoa2* locus by using the nearest marker (rs13476229) as an additive covariate. This did not result in the identification of any additional QTL (Data not shown).

**Table 1:** Plasma total, HDL, and non-HDL cholesterol concentrations in KK × I/L F2 progeny

Concentration, mg/dL	Males (n = 127)	Females (n = 106)
Total cholesterol	180 ± 5	191 ± 5
HDL cholesterol	134 ± 5	123 ± 4
Non-HDL cholesterol*^a^*	46 ± 2	68 ± 3

a *P* < 0.0001 comparing males and females. HDL, high-density lipoprotein.

**Table 2:** Significant and suggestive QTL identified by genome-wide scan of F2 mice

Trait	QTL*^a^*	Chr	Peak cM	95% CI	LOD*^b^*	Nearest marker	Sex	High Strain; Inheritance
HDL cholesterol	*Cq1**^c^*	1	74.7	70.6−81.6	**8.9**	rs13476229	MF	KK, Add
	*−*	2	36.2	21.2−51.2	3.1	rs3718711	M	I, Add
	*−*	3	19.2	17.2−38.2	3.4	rs6324747	F	I, Add
	*−*	4	75.1	60.1−88.6	3.1	UT_4_132.137715	F	KK, Add
	*−*	6	24.8	9.8−44.8	2.9	rs6355719	MF	KK, Dom
	*Hdlq84*	X	38.4	33.4−40.4	**9.3**	rs13483831	MF, M	KK, Add
Non-HDL cholesterol	*Nhdlq13*	1	67.7	56.7−78.7	3.2	rs6185344	MF, F	KK, Rec
	*Nhdlq14*	3	32.4	29.2−35.2	**4.5**	rs6198234	MF	Het, UDom
	*−*	5	42.8	23.8−55.8	2.3	rs3667334	F	KK, Dom
	*−*	7	64.0	50.0−79.0	2.3	rs13479455	F	I, Add
	*−*	14	41.9	19.9−54.9	2.2	rs6156908	F	KK, Add
	*−*	X	38.3	18.4−61.4	3.3	rs13483831	MF, F	KK, Add

QTL, quantitative trait loci; CI, confidence interval; LOD, logarithm of the odds ratio; HDL, high-density lipoprotein.

a QTL were named if they were significant or if they were suggestive but confirmed QTL reported previously.

b Significant LOD scores were determined by 1000 permutations for the autosomes and 16,803 permutations for Chr X and are indicated in bold.

c *Apoa2* is the likely causative gene.

Next, analyses were performed in male and female populations separately to detect QTL controlling HDL and non-HDL levels that may be sex-specific (Figure 1, C and D). For HDL, one suggestive male-specific QTL on Chr 2@36.2cM and two suggestive female-specific QTL on Chr 3@19.2cM and Chr 4@75.1cM were localized. For the Chr 2 QTL, I/L homozygous males showed greater HDL levels in an additive manner compared with KK homozygous males; females did not exhibit this pattern. On Chr 3, I/L homozygous females had greater HDL levels, in an additive manner, compared with KK homozygous females, whereas on Chr 4 KK homozygous females displayed greater HDL levels, in an additive manner, compared with I/L homozygous females. For non-HDL, three suggestive female-specific QTL on Chr 5@42.8cM, Chr 7@64.0cM, and Chr 14@41.9cM were detected. KK homozygous females showed greater non-HDL levels in a dominant manner compared with I/L homozygous females for the Chr 5 QTL and in an additive manner for the Chr 14 QTL. For the Chr 7 QTL, I/L homozygous females showed greater non-HDL compared to KK females in an additive manner. No significant or suggestive male-specific non-HDL QTLs were detected.

A multiple regression analysis indicated that the detected QTL explain 44.9% of the HDL level variation (Table 3). Similar results were found when the analysis was performed in males and females separately, which explained 52.2% and 45.4% of the HDL level variation, respectively. The Chr X QTL contributed slightly more to the variance compared with the *Apoa2* allele in both the combined and male-specific analysis. For non-HDL, a multiple regression analysis could explain only 16.5% of the phenotypic variance in the sex combined analysis but 78.7% of the variance in females alone, which included interactions between two sets of loci and with 25% of the variance contributed by the Chr X QTL (Table 4).

**Table 3:** Multiple-regression analysis for HDL

Sex*^a^*	Chromosome (cM)	df	Variance, %*^b^*	F Value
M + F	Chr1@80.6	2	14.3	28.5
	Chr2@55.2	2	5.0	10.0
	Chr3@19.2	2	1.8	3.6
	Chr6@30.8	2	5.7	11.4
	Chr12@18.1	2	2.7	5.3
	ChrX@35.4	5	15.4	12.2
	Total	15	44.9	
M	Chr1@76.7	2	19.7	25.1
	Chr2@56.2	2	7.6	9.6
	Chr6@27.8	2	3.7	4.7
	ChrX@36.4	1	21.2	53.9
	Total	7	52.2	
F	Chr1@82.6	2	10.6	9.8
	Chr3@19.2	2	9.3	8.6
	Chr4@68.1	2	9.3	8.6
	Chr6@15.5	2	6.4	5.9
	Chr12@2.1	2	9.8	9.0
	Total	10	45.4	

HDL, high-density lipoprotein.

a Regression analysis was performed for the entire population (n = 233) or in males and females separately.

b The variance indicates the percentage of the total variance associated with the respective marker.

**Table 4:** Multiple-regression analysis for non-HDL

Sex*^a^*	Chromosome, cM	df	Variance, %*^b^*	F Value
M + F	Chr1@61.6	2	5.0	6.7
	Chr3@32.4	2	5.6	7.4
	ChrX@35.4	5	5.9	3.1
	Total	9	16.5	
F	Chr1@68.6	2	4.9	8.9
	Chr2@64.1	2	3.6	6.5
	Chr5@43.0	8	13.2	6.0
	Chr7@70.0	6	11.7	7.1
	Chr8@72.7	2	4.8	8.6
	Chr14@47.9	6	10.6	6.4
	Chr19@16.0	2	3.6	6.5
	ChrX@34.4	9	25.0	10.0
	Chr5:ChrX	6	6.3	3.8
	Chr7:Chr14	4	4.6	4.1
	Total	47	78.7	

HDL, high-density lipoprotein; QTL, quantitative trait loci.

a Regression analysis was performed for the entire population (n = 233) or in males and females separately. No significant or suggestive non-HDL QTL were identified in males.

b The variance indicates the percentage of the total variance associated with the respective marker.

### Analysis of *Hdlq84* on Chr X

**Figure 2:**
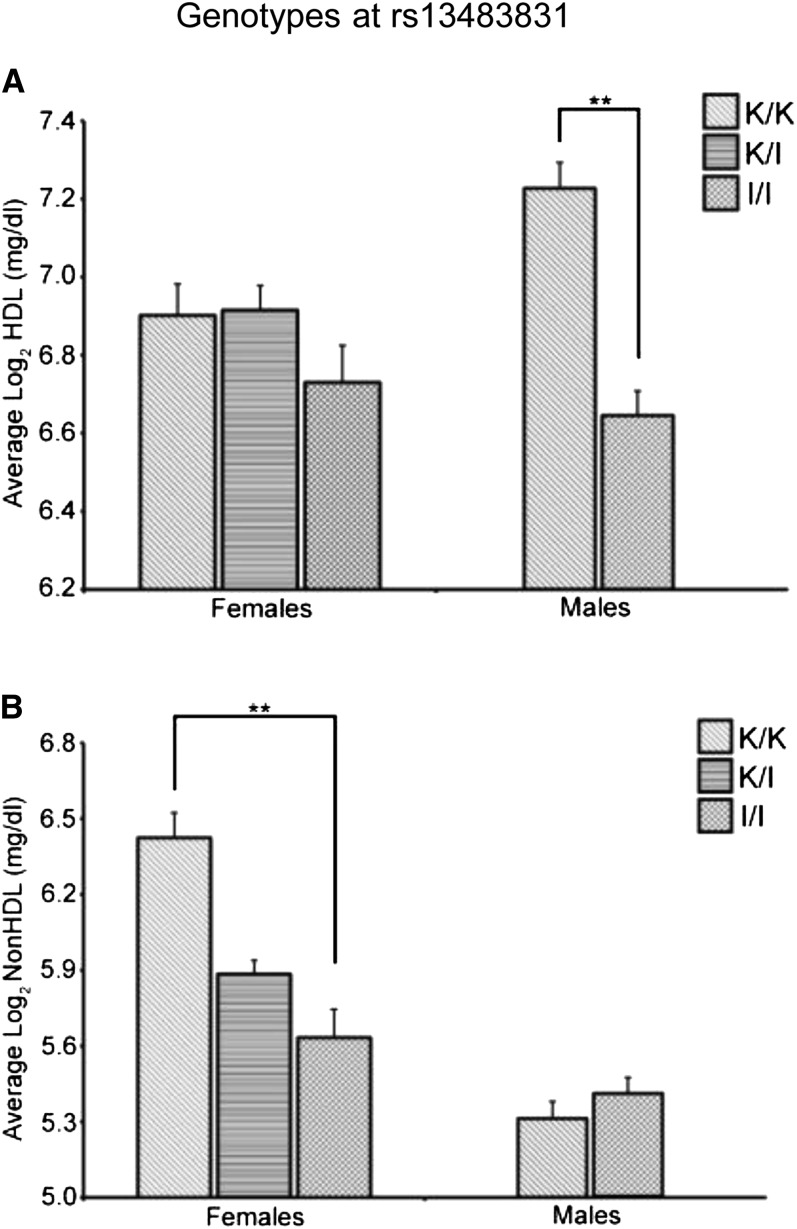
Plasma lipid values based on sex and the genotype at marker rs13483831 (Chr X). (A) HDL cholesterol. (B) Non-HDL cholesterol. K/K denotes mice homozygous for the KK/HlJ allele, K/I denotes females heterozygous for the KK/HlJ and I/LnJ alleles, and I/I denotes mice homozygous for the I/LnJ allele. Error bars indicate SEM. **P < 0.0001.

In the combined analysis, the Chr X QTL (*Hdlq84*) was significant for HDL but only suggestive for non-HDL. However, evaluation of HDL and non-HDL values based on sex show that the Chr X QTL affects males and females differently (Figure 2, A and B). When HDL and non-HDL values were examined based on the genotype for the nearest marker to *Hdlq84* (rs13483831), a significant difference in HDL values was observed based on genotype for males (F_1,125_ = 40.4, *P* < 0.0001) with no difference seen for females. Conversely, a significant difference in non-HDL values was observed based on genotype for females (F_2,103_ = 11.0, *P* < 0.0001) with no effect seen for males.

**Figure 3:**
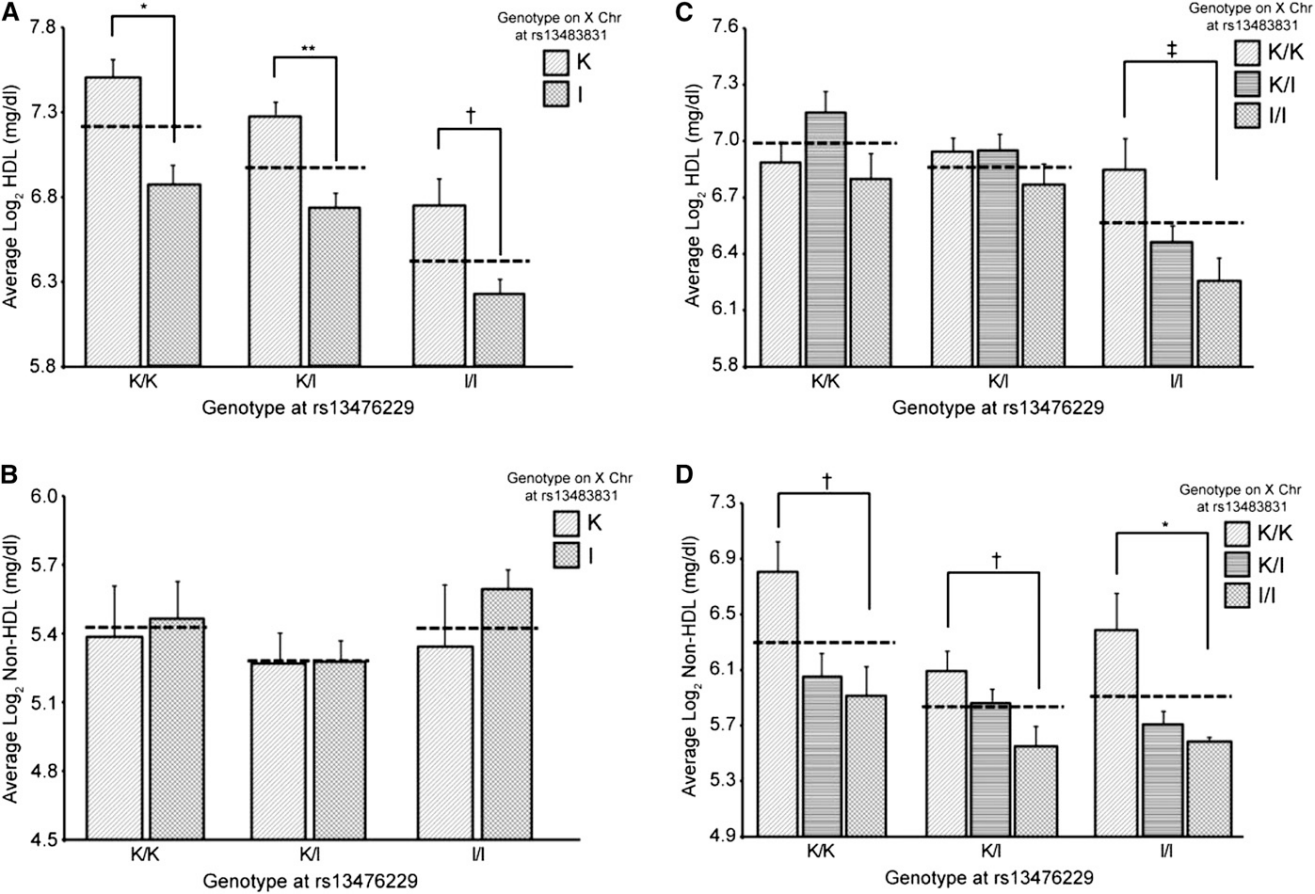
Plasma lipid values based on sex and the genotypes at markers rs13476229 (Chr 1) and rs13483831 (Chr X). (A, B) males; (C, D) females. (A, C) HDL cholesterol. (B, D) Non-HDL cholesterol. K/K denotes mice homozygous for the KK/HlJ allele, K/I denotes females heterozygous for the KK/HlJ and I/LnJ alleles, and I/I denotes mice homozygous for the I/LnJ allele. Dashed lines represent the average values when only considering the genotype at marker rs13476229. Error bars indicate SEM. ^‡^*P* < 0.05, ^†^*P* < 0.01, *P < 0.001, **P < 0.0001.

The Chr X QTL also was analyzed for its affect on plasma lipid values in the context of *Cq1* (nearest marker = rs13476229) by comparing the average lipid values for mice when considering only the genotype at rs13476229 *vs.* mice when considering both the genotype at Chr 1: rs13476229 and Chr X: rs1348483831. For males, mice hemizygous for the KK allele at Chr X: rs13483831 had greater HDL levels than mice hemizygous for the I/L allele compared with only considering the genotype at Chr 1: rs13476229 (Figure 3A). For non-HDL, there was no statistically significant difference in male mice based on genotypes at Chr 1: rs13476229 and Chr X: rs1348483831 (Figure 3B). For females, there was a statistically significant difference in HDL levels for mice that were homozygous for the KK allele at Chr X: rs1348381 *vs.* those homozygous for the I/L allele, but only in those mice homozygous for I/L at Chr 1: rs13476229 (*P* < 0.05; Figure 3C). Females possessing the KK allele at Chr X: rs13483831 had higher non-HDL values, whereas those with the I/L allele had lower non-HDL values compared to the values when only considering the genotype at Chr 1: rs13476229 (Figure 3D).

### Candidate gene identification for *Hdlq84*

**Figure 4:**
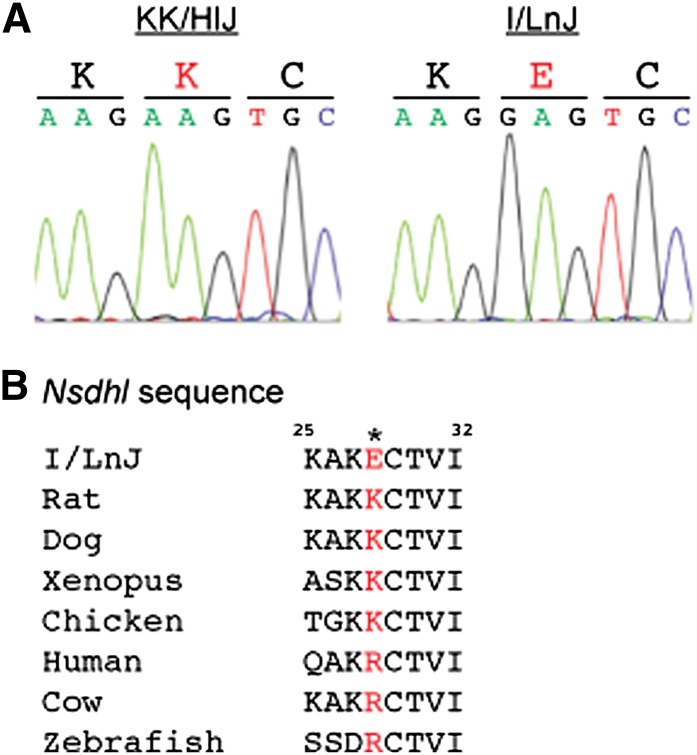
Nsdhl sequence analysis. (A) Sequencing chromatograms for Nsdhl from KK/HlJ and I/LnJ mice showing A->G polymorphism. (B) Nsdhl sequence alignment from various species with the novel polymorphism indicated in red.

Given the magnitude of the influence of the HDL QTL on Chr X, attention was focused on identifying potential causal gene(s). The genomic region (53–80 Mbp, 95% confidence interval) containing *Hdlq84* was examined for genes involved in cholesterol metabolism. Of the 195 genes located in this interval, there is one gene involved in the cholesterol synthesis pathway, sterol-4-alpha-carboxylate 3-dehydrogenase (*Nsdhl*). Mutations in *NSDHL* are associated with the very rare CHILD syndrome in humans, which is an X-linked dominant disorder of lipid metabolism (Konig *et al.* 2000; Bornholdt *et al.* 2005), as well as the recently described CK syndrome (du Souich *et al.* 2009; McLarren *et al.* 2010). Because of the known relationship between NSDHL and lipid metabolism, the coding region of *Nsdhl* was sequenced for both I/L and KK strains (Figure 4A). A unique, nonsynonymous coding polymorphism was found in I/L mice that alters a positively charged lysine to a negatively charged glutamic acid. This polymorphism has not been previously reported, nor is it present in the 18 strains resequenced by the Sanger Center (Keane *et al.* 2011; Wong *et al.* 2012). A positively charged residue is conserved across a number of species, with all species having either a positively charged lysine or arginine (Figure 4B). This unique polymorphism is likely responsible for *Hdlq84*, however further *in vivo* experiments will need to be performed to validate the functionality of the polymorphism.

## Discussion

In the current study, we set out to identify additional QTL that control lipid levels by using KK/HlJ and I/LnJ mice, two strains that are known to differ in their HDL levels. For plasma HDL concentrations, we identified a previously unreported HDL QTL on Chr X (*Hdlq84*) and confirmed two QTL on Chr 1 (*Cq1*) and Chr 6. In addition, we identified one male-specific and two female-specific HDL QTL on Chr 2, 3 and 4. We named the QTL according to the recommendations of the MGI (Maltais *et al.* 2002), thus the QTL on Chr 1 was given the same name as the QTL previously identified in a cross involving KK mice. The QTL on Chr 6 was not named as it has not been previously identified in a cross involving KK or I/L mice and was only suggestive. For non-HDL QTL, we identified two QTL that overlap with the HDL QTL on Chr 1 and X and an additional significant QTL on Chr 3 (*Nhdlq14*). In addition, we localized three female-specific non-HDL QTLs on Chr 5, 7, and 14.

We chose the strains KK and I/L for this study based upon previous data showing a significant difference in HDL level between these two strains when fed a standard diet (Svenson *et al.* 2007). Because of the large difference in basal HDL level in these two strains of mice, we hypothesized this difference would lead to the identification of additional QTL that influence HDL level. On the basis of their differing haplotypes at the *Apoa2* locus (Wang *et al.* 2004), we expected a QTL on distal Chr 1 for HDL level. Even with the strong QTL on Chr 1, we were still able to detect a significant QTL on Chr X that appears to control lipid levels differently in males and females. Adjusting our analysis using either the Chr 1 QTL or the Chr X QTL did not alter our findings or lead to the identification of any additional QTL. This is most likely due to the strength of the signal for each QTL not adjusted for in the respective analyses. Both I/L and KK mice have been used in previous lipid QTL analyses; thus, it is interesting that the Chr X QTL has not been identified. The most likely explanation for why it was not discovered previously is because in the study examining HDL QTL using I/L mice they were fed a high-fat diet (Wittenburg *et al.* 2006), whereas in our study the mice were fed a standard diet.

No previous studies examining HDL have identified significant QTL on Chr X; thus, we focused our attention on identifying the underlying gene for *Hdlq84* by focusing on those genes involved in lipid metabolism. This lead to the identification of a novel nonsynonymous coding difference in *Nsdhl* between I/L and KK strains. We believe this previously unidentified SNP is the causal variant for *Hdlq84* due to Nsdhl being a member of the cholesterol synthesis pathway. This variant has not been identified in human, however other *Nsdhl* mutations are responsible for the X-linked disorders CHILD and CK syndrome. CK syndrome is an X-linked recessive disorder characterized by mild to severe cognitive impairment, microcephaly and cerebral cortical malformations. In addition, some patients suffering from CK syndrome exhibit signs of attention-deficit hyperactivity disorder. Interestingly, I/LnJ mice lack a corpus callosum and also exhibit behaviors consistent with attention-deficit hyperactivity disorder (Magara *et al.* 2000). Further research will be necessary to determine if these characteristics are a result of the polymorphism in *Nsdhl*.

Our study reinforces the importance of taking gender into account when analyzing QTL. Previous studies have identified loci that affect males and females differently (Korstanje *et al.* 2004; Su *et al.* 2008); thus, the identification of QTL that control lipid concentrations differently between males and females is not surprising. However, it is surprising that the same QTL would affect two related traits differently in males and females. Given the strength of the QTL on Chr X, it would not be surprising if there was more than one QTL gene on the X chromosome, however we do believe that *Nsdhl* is a good candidate gene since it is involved in cholesterol synthesis as well as being found on lipid droplets in cells.

This study also shows the limitations with using publicly available SNP data to ascertain candidate QTL genes. Examining publicly available SNP data reveals very few nonsynonymous coding SNPs that differ between KK and I/L mice in *Hdlq84*. In addition, any SNP found to differ between KK and I/L would also have to be the same for all other strains of mice involved in previous HDL QTL studies, as the Chr X QTL has not been previously reported. There are no nonsynonomous coding SNPs currently known that satisfy both of those requirements. In the future, identifying “private” SNPs that are harbored only by a small number of strains is going to require additional sequencing data for the strains used in QTL studies. Already, 18 mouse strains have been fully sequenced (Keane *et al.* 2011; Wong *et al.* 2012), which will aid in identifying any additional “private” SNPs that may be contributing to complex traits. Finally, the fact that we have identified a previously unidentified HDL QTL means that there are likely more genes yet to be discovered that influence lipid metabolism and could serve as additional targets for therapeutic intervention.

## Supplementary Material

Supporting Information
