# Supplementary material for: Identification of a Novel Polymorphism in X-Linked Sterol-4-Alpha-Carboxylate 3-Dehydrogenase (Nsdhl) Associated with Reduced High-Density Lipoprotein Cholesterol Levels in I/LnJ Mice
Source: G3 (Bethesda). 2013 Oct 1;3(10):1819–25. doi: 10.1534/g3.113.007567 (PMC3789806; doi:10.1534/g3.113.007567)
Supplement: Supporting Information [file supp_3_10_1819__index.html]

Identification of a Novel Polymorphism in X-Linked Sterol-4-Alpha-Carboxylate 3-Dehydrogenase (Nsdhl) Associated with Reduced High-Density Lipoprotein Cholesterol Levels in I/LnJ Mice — Supporting Information 

# Identification of a Novel Polymorphism in X-Linked Sterol-4-Alpha-Carboxylate 3-Dehydrogenase (*Nsdhl*) Associated with Reduced High-Density Lipoprotein Cholesterol Levels in I/LnJ Mice

## Supporting Information for Bautz, Broman, and Threadgill, 2013

**Files in this Data Supplement:**

- Table S1 - Data and genotypes for F2 animals (.xls, 532 KB)
